# Building FAIR Functionality: Annotating Events in Time Series Data Using Hierarchical Event Descriptors (HED)

**DOI:** 10.1007/s12021-021-09537-4

**Published:** 2021-12-30

**Authors:** Kay Robbins, Dung Truong, Alexander Jones, Ian Callanan, Scott Makeig

**Affiliations:** 1 Department of Computer Science, University of Texas At San Antonio, San Antonio, USA; 2 Swartz Center for Computational Neuroscience, Institute for Neural Computation, University of California San Diego, San Diego, USA

**Keywords:** Event annotation, Hierarchical Event Descriptors, HED, BIDS, EEG, HED-3G, Neuroimaging, FAIR

## Abstract

Human electrophysiological and related time series data are often acquired in complex, event-rich environments. However, the resulting recorded brain or other dynamics are often interpreted in relation to more sparsely recorded or subsequently-noted events. Currently a substantial gap exists between the level of event description required by current digital data archiving standards and the level of annotation required for successful analysis of event-related data across studies, environments, and laboratories. Manifold challenges must be addressed, most prominently ontological clarity, vocabulary extensibility, annotation tool availability, and overall usability, to allow and promote sharing of data with an effective level of descriptive detail for labeled events. Motivating data authors to perform the work needed to adequately annotate their data is a key challenge. This paper describes new developments in the Hierarchical Event Descriptor (HED) system for addressing these issues. We recap the evolution of HED and its acceptance by the Brain Imaging Data Structure (BIDS) movement, describe the recent release of HED-3G, a third generation HED tools and design framework, and discuss directions for future development. Given consistent, sufficiently detailed, tool-enabled, field-relevant annotation of the nature of recorded events, prospects are bright for large-scale analysis and modeling of aggregated time series data, both in behavioral and brain imaging sciences and beyond.

## Introduction

The FAIR (Findable, Accessible, Interoperable, and Reusable) guiding principles formally articulated by Wilkinson and colleagues (Wilkinson et al., 2016) promote data stewardship and reuse with the goal of enabling scientific evaluation, reproducibility, and discovery. These general guidelines apply not only to datasets, but also to algorithms, tools, and workflows. FAIR is expressed in terms of scholarly digital research objects that can be identified with globally unique identifiers and characterized using metadata selected from formal vocabularies. Importantly, these digital objects should be machine-actionable, meaning that the objects themselves can also provide information with varying levels of detail to autonomous data explorers. Widespread development and adoption of FAIR standards across disciplines is needed to create a robust research ecosystem for supporting interpretable and reproducible science.

### Why HED?

Digital research objects referenced in the FAIR principles are generally larger units — specified at the level of a workflow or a study. Practical implementation of annotation standards and related tool development are necessarily left open to data providers and standards groups. Most current domain-relevant community standards supporting FAIR focus primarily on identification, location, top-level data organization, licensing, and data format specification. While standardization at these levels of detail is crucial, in many disciplines it is not sufficient to support meaningful meta-analysis (combining results or result statistics across studies) and mega-analysis (combining raw data or data features across studies). This is particularly true for ***time-series data*** collected for cognitive neuroscience, psychology, biomechanics, and other brain and behavioral sciences — often in complex, event-rich environments (Boedhoe et al., 2019). Crucially missing from high-level annotation standards focusing on data organization and format are:

- A system for specifying the exact ***nature of events*** (sensory, behavioral, and other) occurring during the experiment and the environmental contexts in which they occur for use in informed data analysis.
- A standardized, machine-actionable system for describing the ***relationship of events*** to experiment context, design and structure.
- A ***mapping of events*** to participant expectation, intent, and task.

We believe the evolving Hierarchical Event Descriptor (HED) system has the potential to capture this information in both human- and machine-usable forms. Current efforts to move HED beyond rudimentary event description to accomplish these goals are termed third generation or HED-3G.

The goal of the HED framework is to facilitate the description, annotation, validation, and extraction of events in time series data. First proposed by UCSD graduate student Nima Bigdely-Shamlo, the HED system has now been under development for more than a decade and has undergone several evolutionary steps as developers and users gained practical experience using it for data sharing, annotation, and mega-analysis (Bigdely-Shamlo et al., 2013, 2016; Bigdely-Shamlo, 2014; Rognon et al., 2013; Robbins et al., 2020). HED was accepted by the BIDS (Brain Imaging Data Structure) governance in 2019 (v1.2.1-) as a standardized method for annotating events in human neuroimaging data (Gorgolewski et al., 2016).

This paper focuses on annotation of events in human electrophysiological experiments involving electroencephalographic (EEG) recording, though equivalent application to magnetoencephalography (MEG) and other brain and behavioral data recording modalities is straightforward. Increasingly, our development of HED-3G has focused on developing the HED infrastructure and enabling extensions that retain and build on this basic infrastructure, while also enabling diverse research communities to include terms needed by their fields or subfields to describe events.

The independence of schema vocabulary from the supporting HED manipulation and computational tools make HED applicable to time series data from other fields for which discipline-specific annotation vocabularies can be built — areas potentially as diverse as clinical neurophysiology, animal behavior, sports medicine, consumer behavior, and stock market economics. Standards for annotation vocabulary and syntax are, however, of no practical use without a readily usable tool framework to use in constructing, reviewing, searching, and computing on the annotated data. Here we describe the HED tools already in place as well as a tool development path needed to facilitate and empower use of HED-3G for event-informed analysis of time series data.

### Events and the Structure of Electrophysiological Experiments

To understand why HED-3G is needed, consider how experiments involving observation of human behavior and physiology are structured with a view to subsequent analysis. Most experiments fall into one of three categories: controlled (laboratory or field) experiments, clinical assessments, or long-term monitoring. Controlled experiments are typically organized in terms of structured participant task-design variables, including sensory stimulation, that are varied during the course of the experiment, typically in a balanced manner, as the measured physiological signals and/or behavioral records are continuously acquired. The behavioral records may also include multiple time-series recordings of participant behavior and environmental (e.g., audiovisual) changes, from which events relevant for data interpretation can be identified during recording or thereafter. Data feature events in clinical assessment records are ‘read’ by clinical neurophysiologists using visual inspection and annotated for clinical purposes using terms such as ‘interictal spike’ or ‘sleep spindle’.

#### **Evolution of Traditional Event-related Paradigms**

Most electrophysiological laboratory experiments continue to use sparse stimulus–response paradigms: perceptually distinct sensory stimuli are presented at recorded times with abrupt onsets and ensuing (and/or preceding) changes in the recorded behavioral and physiological data streams are measured and modeled. Analyses extract data epochs time-locked to selected classes of near-equivalent events that are typically assumed to be associated with consistent brain dynamic patterns, enabling assessment of statistical relationships between type of event and some mean measure or measures of the physiological and behavioral data.

Traditionally, neuroimaging experiments record discrete participant action events, often finger button presses, performed by the participant(s) in response to stimulus presentations as motivated by one or more assigned tasks. Such motor response ‘events’ have typically been analyzed as if they were instantaneous in both time and space (although more complete psychobiological assessment would disagree). However, usefully annotated events need not be limited to stark, sudden onsets of stimuli perceived in isolation by study participants. More fully recorded behavioral responses, elaborated motor actions with measurable temporal and spatial extents, can be captured using body motion capture and eye tracking and/or response collection devices such as touch screens. Such so called mobile brain/body imaging (MoBI) experiments (Makeig et al., 2009) allow examination of brain dynamics supporting a fuller range of natural human embodied cognition. In such paradigms, participant action events of interest may be identified during data collection or during post hoc analysis.

Motor actions or gestures may be modeled by applying relevant measures to data selected and defined in relation to the timing of a sequence of action landmark events. In gait experiments, for example, the exact times of heel-strike and toe-off events in each gait cycle provide information critical for both gait analysis and neuroimaging research (Wagner et al., 2016). In reach-to-touch experiments, the locations and timing of arm/hand movement onsets and offsets, as well as time points of maximum acceleration, velocity, and deceleration are of prime interest (Valevicius et al., 2018). Marking critical points in movement trajectories as sequences of annotated, time-noted events may aid in temporal co-registration and in comparisons across similar or contrasting actions. A similar demarcation of natural speech stimuli by sequences of marked word, phoneme, or other psycholinguistic boundary events may enable temporal co-registration of brain/behavioral data within or across linguistic stimulus and/or utterance categories.

#### **Context-Dependence**

Traditionally, event-related human electrophysiological data collected for cognitive neuroscience has been analyzed by studying its dynamics immediately following (and/or preceding) presentations of a few types or categories of sensory stimuli, delivered in planned and recorded time sequence and having contrasting sensory features and/or task-related significance. Typically, the focus of interest has been on between-category differences in stereotyped data features of category-mean measures immediately following (or preceding) stimulus presentations. However, the primary role of the brain can be viewed as informing, instigating, and evaluating results of behavioral action plans appropriate to the ***evolving context*** of the current moment – taking into account preceding events and their effects on the participant’s evolving expectations of near future event prospects. Flexibility in selecting and organizing context-dependent responses to ever-changing opportunities and challenges is intrinsic to human cognition and a hallmark of general intelligence (Gray et al., 2003). Though this ever-evolving appraisal process can be expected to produce measurable trial-to-trial variations both in cognition and in brain dynamics, such context-dependent variations have so far received far less research attention.

#### **Mega-Analysis**

Most studies do not have enough statistical power to support hypothesis testing at finer-grained levels of analysis than distinguishing among high-level event categories, without consideration of fine contextual differences. A straightforward response to this dilemma is to collect and analyze much larger quantities of data within a single task paradigm, a solution that is often impractical to carry out and also is limiting from a cost–benefit perspective. A powerful alternative is to use new analysis methods to model the diversity of brain dynamics associated with a wider variety of events observed and annotated as occurring in a range of contexts across multiple archived studies recorded using different paradigms. New artificial intelligence (AI) methods, increasingly being applied to diverse data collections, show the possibility of such approaches being able to reveal new, more detailed information about human cognition, behavior, and health, and their supporting brain dynamics in a wider variety of circumstances.

To do this, however, requires having detailed descriptions of behavioral, environmental, and task events occurring during the recordings. Meta- or mega-analysis across event-related studies requires detailed specification of events as well as information about spatiotemporal context − the participant environment, recording parameters, event history, control variables, and task behavioral imperatives (Costafreda, 2009). Additional challenges (as well as opportunities) are posed by the recent trend towards studying human natural cognition in more general and natural circumstances − recording conditions in which participants listen to fluent speech, watch movies, perform ambulatory tasks in virtual, augmented, or actual reality laboratory environments, interact in some ways with other participants, or even participate in real-life activities.

The spatiotemporal complexity and variety of electrophysiological and biomechanical dynamics (even within a typical simple oddball stimulation paradigm) make full interpretation dependent on knowing the basic nature, timing, and personal significance of the then-current participant sensory experience or behavior, as well as participant intentions. The importance and complexity of the problem of event description and annotation across a diversity of data recording contexts thus requires development of a dedicated tools and language framework, a set of needs that the HED development effort is attempting to fulfill.

#### **Clinical Assessments**

Electrophysiological time series recordings performed for clinical assessments or long-term state monitoring are typically stored with detailed information about patient medical state in addition to standard subject demographic metadata such as age, gender, and handedness. In current clinical practice, events of interest are patterns in the recorded data that are detected and assessed visually by experienced clinicians for a range of known clinical signs. Mega-analyses across studies in projects to develop biomarkers for diagnostic applications need machine-actionable versions of this complex clinician-added metadata. In the HED-3G model described below, the base HED schema vocabulary can be extended to include clinical assessment terms and events by creating a HED library schema for this purpose.

### Community Standards for Electrophysiological Time Series Data

The diverse annotation requirements of human electrophysiological experiments make development of useful community standards quite challenging. The BIDS (Brain Imaging Data Structure) community (https://bids.neuroimaging.io) and its imaging modality subgroups are making a sustained and increasingly successful effort to implement FAIR standards (Gorgolewski et al., 2016; Niso et al., 2018; Pernet et al., 2019; Holdgraf et al., 2019) at the level of data (and metadata) formatting and file organization, with emphasis on simplicity and efficiency. BIDS now has a large community of active users/developers and has quickly become the de facto standard for organizing human neuroimaging data. BIDS has incorporated support for a variety of imaging modalities as well as auxiliary behavioral and other physiological measures. Many of the major brain imaging software tools and archives now support or will soon support data sharing using BIDS. Work continues on BIDS specifications for derived data as well as for auxiliary streams such as eye tracking data. The BIDS community also supports development of some standardized processing pipelines (as containerized ‘BIDS Apps’) for fMRI and other types of data. Public data repositories such as *OpenNeuro* (https://openneuro.org) and computational portal sites such as *Brainlife* (https://brainlife.io) and *NEMAR* (https://nemar.org) now organize their shared data in BIDS.

In 2019, BIDS (v.1.2.1) adopted HED as its event annotation standard to the extent that it allows (but does not yet require) BIDS users to incorporate HED event annotations. Originally, BIDS (2016–19) only allowed users to include home-grown “event codes” as additional columns in the *_events.tsv* files that describe events occurring during the recording. BIDS now also allows users to document the meaning of these event codes, as well as the meanings of other columns in associated _*events.json* files, using both HED terms and free-form text descriptions. BIDS will also soon support HED annotation in other metadata files such as participant information (*_participants.tsv*) or information about the data acquisition runs and sessions (*_scans.tsv*). HED tools will automatically use these TSV and JSON files to assemble full HED string event annotations for analysis.

### Sharing Time Series Data to Enable Across-study Analysis

BIDS and the inclusion of HED event annotations into BIDS are important steps in establishing open standards for analysis-enabling event annotation. However, much practical work is still needed on both study structure and event annotation to achieve effective data-sharing and to enable meta/mega-analysis of neurophysiological and other data that models interactions between brain dynamics and its ever-evolving cognitive context. This gap in readiness for data sharing is particularly evident for research using high-resolution time series recordings of brain electromagnetic field fluctuations − electroencephalography (EEG), magnetoencephalography (MEG), electrocorticography (ECoG) and other intracranial recording technologies (iEEG). In such experiments, event-related brain or brain/body dynamics are the central focus of research interest, yet datasets released under current standards typically lack the critical information needed to document the complexity and essential details of noted events that is needed for full analysis.

The barriers to achieving the goals of effective data sharing of neurophysiological and other event-related time series data are related to both articulation (means) and motivation (motive):

- ***Articulation*** barriers are technical; the event annotation system must be sufficiently expressive to adequately retain the information needed for within- and between-study analyses, using standard tools and vocabulary rather than local laboratory jargon (e.g., *Target*) or intrinsically meaningless designations (e.g., *Event type 13*). Further, the annotation system must be capable of capturing the structure of the experiment, providing mappings of structure information to the events and to the data in a form that is both readily human-comprehensible and machine-actionable. Annotations should also be able to document task-engendered relationships of task events to one another, including relationships between sensory and behavioral events.
- ***Motivational*** barriers are even more challenging, since the needed level of annotation rises well beyond current standards for data-preservation and data-sharing. The annotation process must offer researchers clear value added, ideally enabling researchers who more thoroughly annotate events to then make use of tools that extract useful information from the data. These tools may also give the researchers meaningful information about the relationship of the data to existing archived data collections from related but not necessarily identical experiments. Above all, the annotation process must not prove so difficult to perform that the cost of building the annotation exceeds its perceived benefits.

Work on the HED system over the past decade has proceeded with an eye towards overcoming both the articulation and motivation barriers to accurately documenting recorded bio-behavioral data to enable mega-analysis in human electrophysiology and related fields. This paper describes the evolution of HED (“HED structure, history, and status” section), and how the current development of HED-3G enables progress towards these goals (“HED-3G: Annotation to inform advanced analyses” section). Finally, “HED now and future: A roadmap forward” section presents a roadmap of steps needed to make routine, adequate annotation of both shared and newly collected event-related neuroimaging data a reality.

## HED Structure, History, and Status

The HED-3G system described here consists of: 1) a hierarchically-structured schema vocabulary and syntax that allows events, experiment design, and control variables to be annotated and identified; 2) a set of software tools to facilitate data annotation, validation, search, and analysis. Individual HED event descriptors (HED tags) are text path strings grouped into shallow tree structures to allow meaningful organization of related terms into easily searchable subcategories. Full event annotations (HED strings) are comma-separated lists of HED tags and parenthesized HED tag groups. Full HED-string annotations can be validated for compliance against a specified vocabulary – the base HED schema, possibly extended by one or more new HED library schemas that define and organize additional descriptive terms relevant to a particular research field or subfield. HED-tag text and prefix matching are easily implemented, allowing effective event search, extraction, and accumulation for analyses within or across available data sets.

The original HED specification and supporting tools have undergone several structural revisions as first users gained experience with annotating data. We now briefly describe those reorganizations and why these changes were necessary to support the evolution of HED into an effective annotation, search, and analysis system.

### First Generation HED: Strict Hierarchy

The initial HED system (HED-1G) was initially deployed in 2011 to support annotation of events in HeadIT (https://headit.ucsd.edu), an early public repository of EEG data hosted by the Swartz Center for Computational Neuroscience at UCSD as part of an infrastructure to enable searches through archived data for event-related source EEG patterns (Bigdely-Shamlo et al., 2013). Early versions of HED-1G terminology were partially based on CogPO (Turner & Laird, 2012). Event annotation in first-generation HED was organized around a single term hierarchy whose base was *Time-Locked Event*. Users could extend the HED schema hierarchy at its deepest (leaf) nodes to provide additional detail (https://www.hedtags.org/display_hed.html). Several EEG studies were successfully annotated for public distribution and first analyses applying HED tools to the repository datasets were demonstrated.

Unfortunately, once developers began annotating more complex datasets, they encountered a fundamental design flaw illustrated in the following example. (For simplicity in this and following examples, we focus only on annotation of stimulus shape and color, although HED also supports descriptions of further details such as size, location, task role, and expected participant response.) The HED strings represent the annotation for a sudden-onset event. The actual event onset time value and the manner in which this onset time is associated with the corresponding annotation depends on the dataset representation as discussed later.

**Figure:**
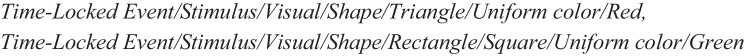
**Example 1. A first-generation HED annotation** of a presentation of a visual stimulus consisting of *both* a red triangle and a green square.

HED-1G had no mechanism for designating some tags as modifying other tags. If red squares and green rectangles were also possible, the terms *Red* and *Green* would have to appear in two places in the schema hierarchy. If annotators wanted to include some color detail in annotating a participant response action event (e.g., to describe the event in which a participant presses a red button), they would also have to add the same color terms to the *Time-Locked Event*/*Response* branch of the purely hierarchical HED-1G schema. In this way, attribute terms such as *Red* and *Green* in Example 1 proliferated throughout the schema hierarchy, resulting in an explosion of replicated terms. Further, reversing the order of *Uniform color/* and *Shape/* in the tags of Example 1 would give equally valid annotations, making testing for tag equivalence difficult.

### HED-2G: Orthogonality and Abstraction

This “many *Reds*” problem of first-generation HED demonstrated that adjectival information such as color and location should be treated as descriptive properties or item *attributes* rather than categorical item subtypes. The HED working group realized that adjectival *Attribute* tags (including *Red* and *Green* in Example 1) should be separated from nominative *Item* tags representing objects (such as *Square* and *Triangle*). *Attribute* tags should also be separated from tags for higher-level event concepts, for example those distinguishing *Stimulus presentation* from *Experiment control* events. This insight led to a major redesign focused on removing ambiguity and improving expressiveness, while limiting prolixity (Bigdely-Shamlo et al., 2016).

The idea of the redesign was to group independently applied (“orthogonal”) terms and concepts into separate hierarchies, thus making HED strings structured as tag heterarchies (collections of hierarchies) rather than as a single hierarchy. In second generation HED (HED-2G), the top-level hierarchies were (roughly) nouns (*Event*, *Participant*, *Paradigm*, *Experiment context*), adjectives (*Attribute, Sensory presentation*), or verbs (*Action*). Further, HED-2G syntax allowed arbitrary levels of nested parentheses to enable grouping of attributes (adjectives) with the items (nouns) they modify. For an expandable *HTML* view of second-generation HED, see (https://www.hedtags.org/display_hed.html).

**Figure:**
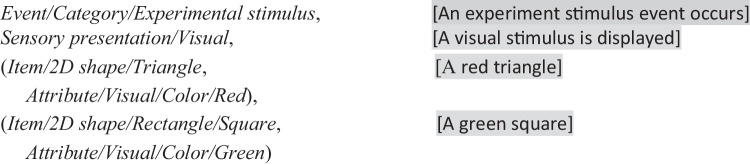
**Example 2. Second-generation HED annotation **for a visual stimulus presentation of a red triangle and a green square. [Note that, here and below, text in square brackets is didactic commentary, not HED syntax.]

HED-2G included many other refinements, including a BIDS-compatible specification and validation of unit classes associated with use of numeric values.

### HED Tools for HED-2G

During the evolution of second-generation HED, several software tools were developed to improve HED usability (https://github.com/hed-standard) and to assure the independence of HED validator tools from the particular version of the HED vocabulary schema used for the annotation. This **separation of implementation from interface** allows any appropriately formatted controlled vocabulary to be validated without changing the validation tool infrastructure.

A user-friendly GUI, *CTagger* (community tagger), initially developed for HED-1G (Rognon et al., 2013), was further enhanced for HED-2G. *CTagger* is platform-independent and can be run as a standalone application or as a plug-in for EEGLAB (Delorme & Makeig, 2004). A *pop_hedepoch* function for EEGLAB allowed users to select EEG or other time series data epochs time-locked to selected events whose HED strings included any desired combination of HED tags. HED-2G validation tools in MATLAB and Python, as well as web-based validation tools, were also developed.

### First Applications to EEG Mega-Analysis

A large-scale multi-study mega-analysis of data across 18 different studies whose events were annotated using second-generation HED (Bigdely-Shamlo et al., 2019a, b; Robbins et al., 2020) demonstrated a fundamental result, that across studies, time-locked features of trial-averaged event-related potentials (ERPs) and event-related spectral perturbations (ERSPs) associated with HED strings containing the same HED tags were significantly more similar than event-related averages of epochs time-locked to events with fewer tag similarities. Without the work performed to add detailed and consistent HED tag annotations to these studies’ event records, these cross-study comparisons would have been highly laborious if at all feasible.

### The Transition to HED-3G

Our initial experience with large-scale, automated analysis exposed both the strengths and limitations of HED-2G annotation. In late 2019, motivated by this understanding, we began the HED-3G redesign of HED. Initially, our efforts focused on cleaning up a vocabulary that had grown by accretion rather than through strategic planning as more datasets were annotated. When it became apparent that HED-3G had the potential to seriously address the outstanding issues raised in the “Introduction” section, our HED-3G working group began restructuring its development efforts.

The fundamental advance of HED-2G was recognition of the role of orthogonality in vocabulary design. Multiple levels of parentheses were also introduced so that modifiers could be properly associated with the items they modify during analysis. The most important structural advances thus far in HED-3G are ***unique mapping***, the addition of ***user definitions*** and ***organizational tags***, the formalizing of the concept of ***event duration*** and overlapping ***event context***, and the introduction of subsidiary ***library schemas***, The next section explains how these changes contribute to the goal of comprehensive machine-actionable annotation of events, while “HED now and future: A roadmap forward” lays out the roadmap for future development.

## HED-3G: Annotation to Inform Advanced Analyses

The new base HED-3G schema specification (current version, *8.0.0*) clarifies and simplifies the structure of the upper-level HED vocabulary schema to better-support annotation and readability. It also increases the precision of the HED syntax and expands the scope of the base HED schema to better support specification of experiment design and structure as well as participant task, intent, and expectation, although more fundamental work on these is still needed. Why is this additional information an essential part of event annotation? Because the aspects and attributes of events that are most important to document and apply in subsequent analysis are their relationships to participant task, intent, and expectation in the current temporal context, which in turn is intrinsically connected to experiment design and structure.

### **Unique Mapping**

A key new concept in HED-3G is its **unique mapping** rule. In HED-3G individual terms (node names in the schema trees) used in HED tags may appear in no more than one place in a schema. While this requirement may somewhat complicate the HED schema-design process, it offers great improvements in usability for HED users. Users can now just use single (leaf) node names instead of complete tag path hierarchies during annotation; HED tools can then expand the ‘short form’ annotations to full tag paths.

### **User Definitions**

Many research labs develop shorthand ‘lab jargon’ terms to refer to event types used in their experiments (‘targets, ‘standards’, etc.). Such descriptions are not standardized across laboratories and omit many details crucial to efficient cross-study search and analysis. HED user definitions allow users to give detailed definitions of lab jargon terms once, early in the annotation process, thereby retaining the mnemonic advantages of jargon for the annotator, while avoiding its vagarity in shared or archived data.

### **Event Duration and Context**

Another key HED-3G advance is the introduction of comprehensive mechanisms for handling of events with different durations and overlapping time boundaries. The need for this capability is motivated by the important ***context*** sensitivity of brain dynamics*,* essential for enabling the human brain to adapt behavior and experience flexibly in light of ever-changing needs, threats, and opportunities. HED-3G definitions and organizational tags play a crucial role in supporting these mechanisms.

### **Library Schemas**

HED-3G also introduces the concept of subsidiary **HED schema libraries** that expand the HED base schema tag vocabulary by providing terms for describing events needed by particular user communities (clinical practice, language research, etc.). Though extensive, this formal schema reorganization does not significantly impact the use of HED in BIDS, as the HED validation tools have been built to validate against any specified HED schema.

### Reorganization of the HED Tag Vocabulary

**Fig. 1:**
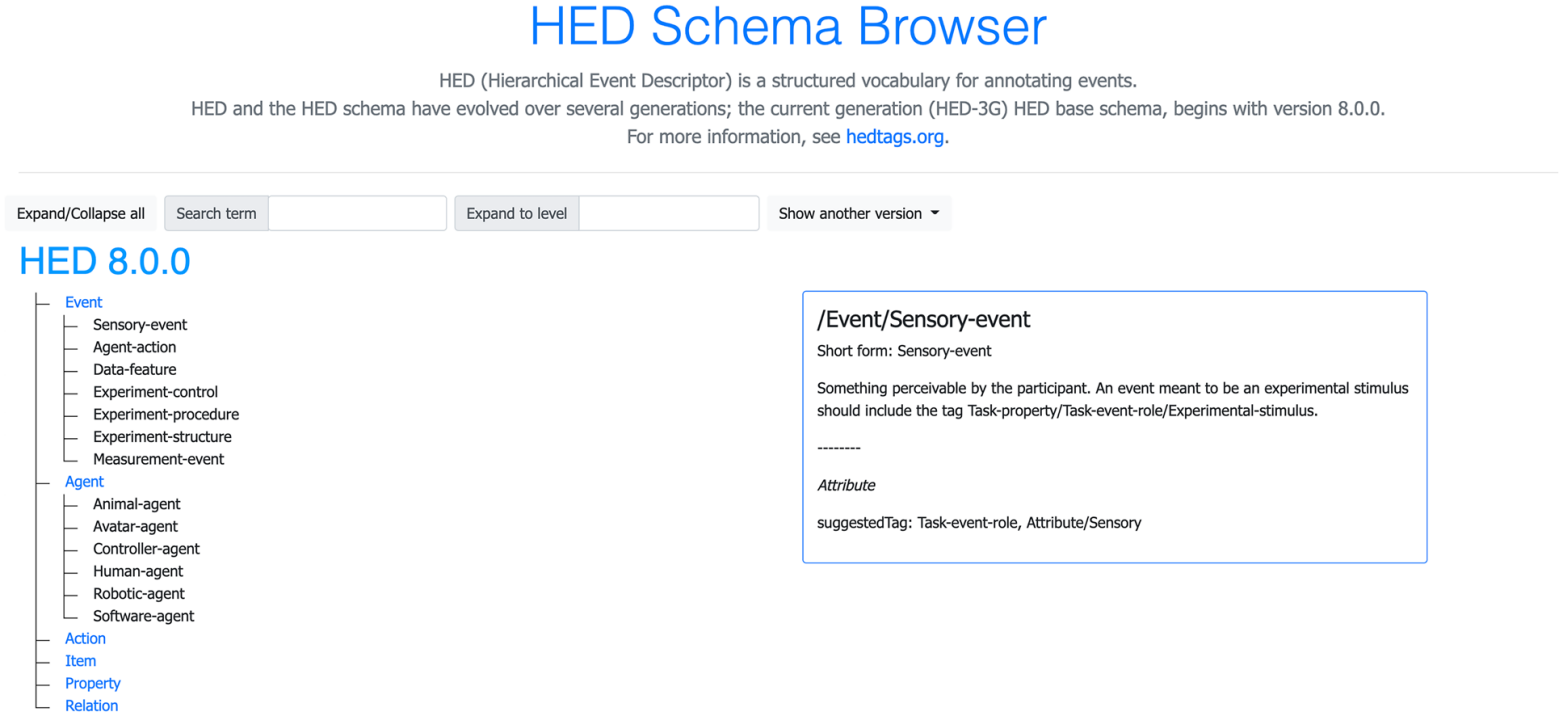
The HED schema browser provides an expandable HTML view of the schemas that are available in the official *hedxml* repository. Users can expand or collapse the view for ease in navigation. The schema trees are on the left. The right box shows the details of the entry over which the viewer’s cursor is hovering. HED-3G and previous generations of HED are available at (https://github.com/hed-standard/hed-specification). This figure is a screenshot of the HED-3G expandable viewer (https://www.hedtags.org/display_hed.html)

As the HED-2G vocabulary expanded, some branches of the HED-2G schema hierarchy became quite deep and detailed, while other branches remained relatively bare, making search through the tag term forest frustrating and posing a significant usability barrier. A more compact and easily searchable HED schema format was needed to improve HED system effectiveness and usability. Figure 1 displays the redesigned schema using the new online HTML schema browser that allows users to explore any available version of the HED schema with expandable or collapsible views. In the fully expanded view users can use the browser find-in-page search features to find particular items.

Computer menu usability guidelines suggest limiting sub-categories to fewer than 10 items (Carliner, 1987), ideally 3 to 7. As part of the redesign, the HED-3G vocabulary was therefore significantly reorganized for clarity into the following six top-level categories (with numbers in parentheses indicating the number of second-level categories): *Event(7)*, *Agent(6)*, *Action(5)*, *Item(4)*, *Property(7)*, and *Relation(5)*. This organization reflects the trade-off between hierarchy balance and depth under the constraints of orthogonality. In addition to vocabulary reorganization, the schema description and purpose of each tag are being improved, and *suggestedTag* and *relatedTag* attributes are being added for individual tags. In Fig. 1, *suggestedTag* value *Property/Task-property/Task-event-role* is displayed in the details box on the right when the hovers the cursor over an element in the schema tree on the left. These tags will allow tool-builders to easily incorporate hints to assist users during annotation, review, and analysis. The planned addition of an ID to each node in the schema hierarchy will allow future development of databases of examples relevant to tags as well as links to external information sources and ontologies.

### Unique Mapping and the Introduction of Short Forms

In previous versions, HED strings were always built, displayed, and reviewed in fully elaborated format. In HED-3G, a full path annotation is now referred to as a node’s **long form**. However, when researchers wish to detail the nature of not yet annotated events or review how events have been annotated, full long-form HED strings can be difficult to read quickly. If the individual nodes in a schema hierarchy have unique names, it is easy to expand any node name or its partial path into its full path. The use of any partial path from a schema’s node-name to its schema tree root is referred to as a **short form**.

HED-3G requires that all tools from validation through analysis support short form and provides library functions in Python, JavaScript, and MATLAB to support translation. HED-3G **short form** syntax compresses the HED string syntax to enable quick composition and review. The concise representation is designed to make HED-3G annotations easier to read, write, and review than their complete long forms as illustrated by the following example.

**Figure:**
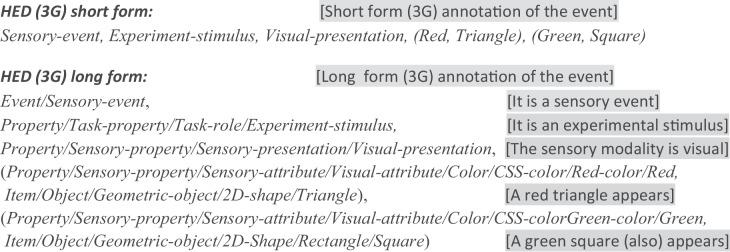
**Example 3. Full long form and compact short form** of a HED-3G annotation of the same event as in Examples 1 and 2, in which a red triangle and a green square are shown to the participant. Again, stimulus size, duration and positioning details are here omitted for brevity.

The two HED string annotation versions in Example 3 above describe the same event as Examples 1 and 2. Their difference in ease of comprehension is evident, yet the full long form can be automatically derived from the short form because it is built on a base HED schema (8.0.0ph) that satisfies the HED-3G unique mapping rule. Uniqueness allows HED tools to present and translate HED strings interchangeably between long and short forms. For added clarity, the composer can include as much of the relevant tag prefix as desired (for example, using *Sensory-presentation*/*Visual-presentation* rather than just *Visual-presentation* above).

Notice that although the HED-2G and HED-3G HED strings in Examples 2 and 3 are equivalent, the HED-3G schema has been somewhat reorganized to satisfy uniqueness and orthogonality. The HED-3G string *Event* type (*Sensory-event* in Example 3) indicates that an environmental sensory event has occurred in the participant’s field of view. In contrast, HED-2G directly specifies that an *Experiment-stimulus* has occurred (Example 2). Since the same sensory event can be a stimulus in one task and not in another, this organization makes it difficult to annotate sensory events in a consistent manner.

In HED-3G, the relationship of the sensory event to the intent of the experiment (recording the role of the event in the experiment structure) is specified using further tags. This separation in HED-3G between sensory events and experiment design is based on the recognition that brain and behavioral dynamics are affected by sensory input in complex ways that are highly dependent on the participant's perceived significance of the event within the currently evolving context.

### Expanding HED with Library Schemas

A major shortcoming of HED-2G was the tendency for users, when faced with a new concept, to add overly-specific terms and jargon to the base schema – for example, adding musical terms to tag events in music-based experiments, video markup terms for experiments involving movie viewing, traffic control terms for experiments involving virtual driving, and so forth. Clinical fields using neuroimaging also have their own specific vocabularies of terms for noting data features of clinical interest (e.g., ‘*seizure’*, ‘*sleep stage IV’*). Including all possible research-area-specific terms in the base HED schema would quickly make the vocabulary wholly unwieldy and practically unusable. In building the base HED-3G schema, therefore, we have tried to remove terms with an overly-specific field of use.

To accommodate the annotation needs of specific research and clinical subfields, HED-3G introduces **HED library schemas**. To use a programming language analogy: when programmers write a C or Python module, its code does not become part of the standard C or Python library. Instead the module is embedded within an application library that is included when needed by an application. Similarly, in addition to the base HED-3G schema, users may use tags from one or more HED library schemas to describe events in their data. HED library schemas must conform to the same syntax as the base HED schema, and should follow four basic rules:

1. Schema terms should be readily understood by most users (*Clarity*).
2. Within a library schema, every term *must* be unique (*Uniqueness*).
3. Terms used independently *must* be in different sub-trees (*Orthogonality*).
4. Term hierarchies should have a moderate number of subcategories at each node, ideally in the range 3 to 7 subcategories (*Structural sparsity*).

As with C or Python libraries, we anticipate that many different HED schema libraries may be defined and used in conjunction with the base HED schema to annotate details of events in experiments designed to answer questions of interest to particular research or clinical communities. Since it would be impossible to avoid naming conflicts *across* schema libraries built in parallel by different user communities, HED-3G supports distinct schema library *namespaces*. Users can define a local namespace name within their file and associate the identifier with an external library schema. Annotations identify the source of terms defined in a specific HED library schema by prepending namespace designators (using format, *Library_identifier:Tag-term)* to use the *Tag-term* term from the library schema designated by its brief library namespace identifier.

The first HED library schema, now under construction, will implement the standardized SCORE vocabulary used by clinical neurophysiologists and neurologists worldwide in reporting their visual (and/or software-aided) evaluation of clinical EEG data (Beniczky et al., 2013, 2017). The development of a HED library schema for SCORE will allow archiving of annotated clinical EEG data in BIDS or other formats that accept HED annotations, hopefully enabling large quantities of such data to be accumulated for clinical and basic exploration and discovery using now rapidly advancing machine learning methods.

The SCORE library schema will be the first to be included in a planned central HED library schema registry (https://github.com/hed-standard/hed-schemas). Although private HED schemas may also be used, annotations of shared data using registered and openly shared HED library schemas will be of value to more users for more purposes, and will thus be encouraged.

### Definitions, Experimental-structure, and Time

HED-3G also introduces a number of *structural* enhancements that allow annotators to capture richer information about experiment events in ways that are both human- and machine-actionable. This information includes the nature and structure of the control variables, the temporal organization of the recordings, and detailed contextual information describing the conditions under each event occurs. HED-3G introduces user-developed ***Definition*** tags not only to facilitate tag reuse and minimize tag repetition, but also as the foundation for annotation of complex structure and temporal evolution.

*Definition* tags allow users to use terms they normally use in the laboratory to describe their data, while mapping them into standardized annotations appropriate for sharing. Users specify a named *Definition* tag associated with a tag group of elaborative HED tags as shown in Example 4. The defined name can then be used to represent that group of tags during annotations. HED tools automatically handle the translation during validation, event-related data search, and analysis.

**Figure:**
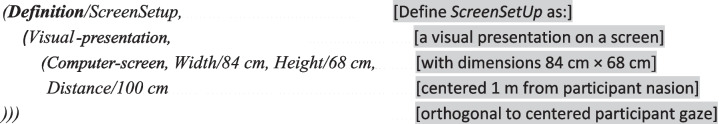
**Example 4. Define** ***ScreenSetup*** **to represent the experimental setup used to present visual stimuli.**

Once *ScreenSetup* is defined, the tag *Def/ScreenSetup* can be used in annotations to avoid repeating these screen description tags in every screen-presented visual event. The ‘*Def/’* prefix is required in the annotations to allow the HED validator and analysis tools to identify *ScreenSetup* as an unexpanded definition name. During analysis, tools will insert the entire definition in place of *Def/ScreenSetup* to create a fully-elaborated HED string annotation for each event. However, the *Definition/ScreenSetup* tag in the definition will be replaced by *Def-expand/ScreenSetup* so that the inserted tags retain an association with the definition but are not confused with the definition, itself. In practice, a lab-specific set of definitions can be built and used for tagging all relevant lab data sets, further speeding annotation of new and existing data.

#### **Annotating Event Duration**

Events without explicit temporal extent (e.g., onset, offset or duration) are modeled as instantaneous (i.e., occurring at a single instant). In HED-3G, the ability to give tag groups explicit *Definition* names also provides a foundation for specifying the temporal extent (time span or temporal scope) of ‘enduring’ events having measurable temporal extent. Tagging an enduring event’s temporal extent explicitly allows HED tools to support analysis of events modeled (more flexibly and often, realistically) as processes unfolding through time. For example, in a reach-to-touch gesture in a touchscreen task or in a step cycle during a treadmill walking task, each participant action has an appreciable duration within which various critical stage events may be annotated for analysis (e.g., stimulus or movement onset, offset, points of max acceleration or velocity, etc.).

Enduring events may be indicated explicitly using pairs of instantaneous ***Onset*** and ***Offset*** events linked to each other by a common tag-group definition name. A defined name grouped with an *Onset* tag marks the beginning of the enduring event. The end of the enduring event occurs either when the defined name is grouped with an *Offset* tag or when it is grouped with an *Onset* tag. All tags in a tag group containing a *Duration* or *Onset* are assumed to apply throughout the enduring event. Tags not appearing in a tag group containing *Duration* or *Onset* are assumed to apply only to the marked instant. During analysis, HED tools keep track of which enduring events are ongoing at each moment and add ***Event-context*** information to the HED string for each event, as detailed below.

An event string that is grouped with a ***Duration*** tag also represents an enduring event. The onset (i.e., the beginning of the time span) of this enduring event is the time of the event whose annotation contains the *Duration* tag group. The enduring event’s offset is not recorded explicitly as a separate event, but calculated by adding the duration value to the onset time. Multiple tag groups containing *Duration* tags with different duration values may appear in the same event annotation.

#### **Enduring Events and Experiment Design**

An important addition to HED-3G is the capability to embed analysis-ready annotation of experiment design and task organization via enduring events. This embedding is accomplished using the HED-3G organizational tags ***Recording***, ***Task***, ***Condition-variable*,** ***Time-block,*** and ***Experimental-trial*** in conjunction with enduring events.

The *Recording* tag is a convenient organizational tag for grouping metadata and setup information relevant to the entire recording. The *Recording* tag is often associated with an enduring event spanning the entire recording. We anticipate developing tools tailored to specific dataset organizations such as BIDS that automatically gather relevant metadata and setup information stored in auxiliary files and insert this information in tagged form as such an enduring event.

A task is a limited set of structured and, typically, instructed mental and/or physical activities performed by the participant during the recording; usually these are integrally related to the planned data analysis. The *Task* tag is generally a top-level organizational concept used to organize the annotations of these activities and their relationship to recorded events.

A condition variable is an aspect of the experiment that is set or manipulated during the experiment to observe an effect or to control bias. Condition variables are sometimes called independent variables or contrasts. The *Condition-variable* tag is used to organize the annotations that describe these conditions. Often an *Condition-variable* is used as part of the annotation of an event to indicate that the specified experimental condition was in effect during that event.

Many electrophysiological experiments are organized into distinct blocks of contiguous time interspersed with breaks for participant relief and setup changes. The *Time-block* tag organizes tags used to annotate what is happening during such a block. *Time-block* tags are usually associated with enduring events marking the temporal span of the blocks.

In many electrophysiological experiments designed for event-related analysis, a specific set of events occurs in sequence (e.g., a stimulus presentation followed first by a behavioral response and then by some sensory feedback), and the contiguous data segment containing this sequence is extracted for analysis. The contiguous data block is sometimes referred to as an experimental trial. The *Experimental-trial* tag organizes annotations associated with an experimental trial. The *Experimental-trial* tag may be associated with an enduring event. Another use of the *Experimental-trial* tag is to group events associated with a given trial. For example, a tool could automatically identify which events are part of each trial based on a task specification. The tool could then insert the tag *Experimental-trial#*, where # is the trial number, in the HED annotation of each event.

**Fig. 2:**
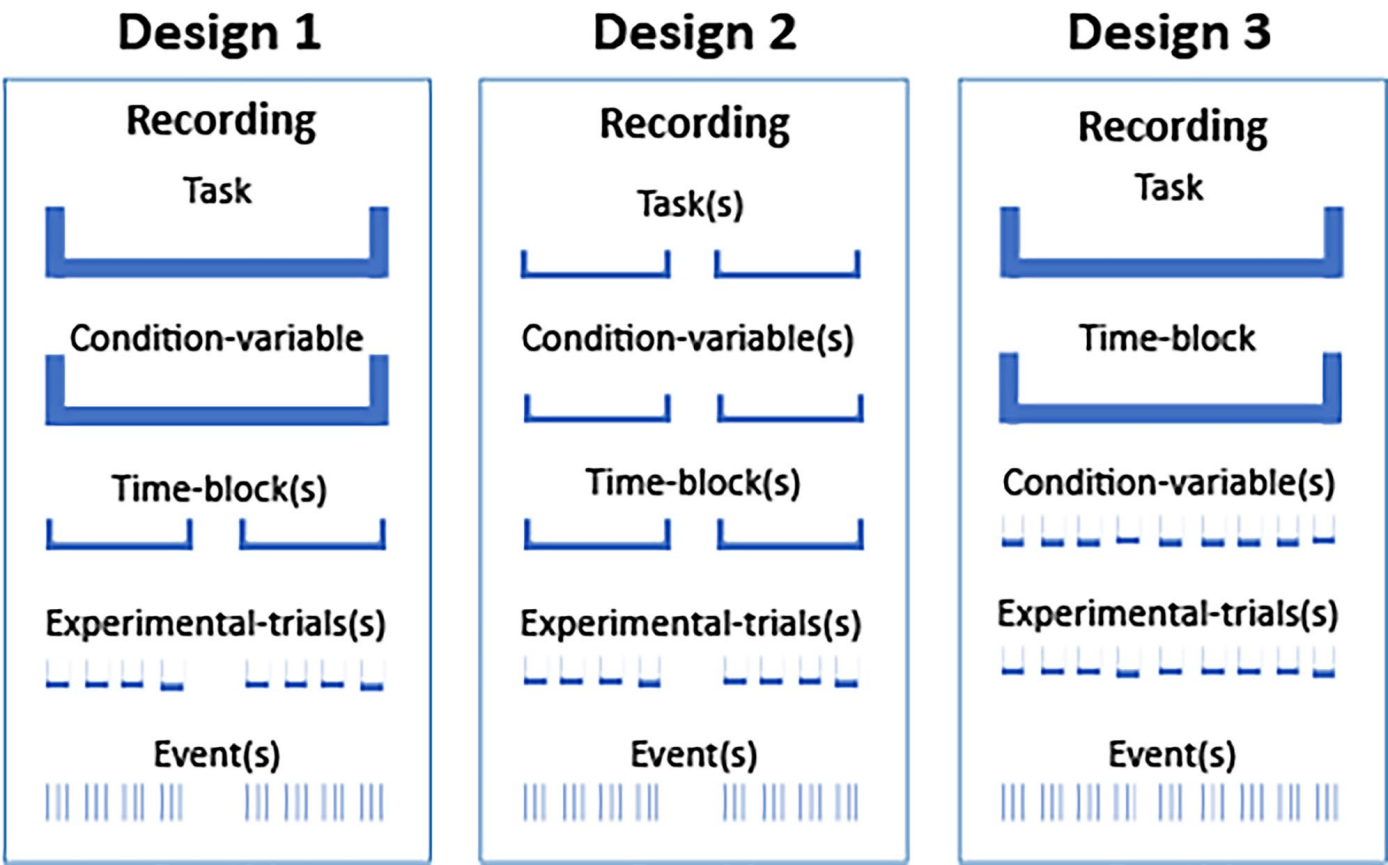
A schematic of the three experiment designs described in Example 5. In Design 1 (left) the participant performs a single *Task* under a single *Condition-variable* in the recording. The recording includes two *Time-block* elements, each containing multiple experimental trials. The Design 2 (center) recording period also has two *Time-blocks*. Each participant performs one experiment *Task* under a single *Condition-variable* in each *Time-block*, counter-balanced by *Time-block.* The Design 3 (right) recording has a single *Task* and *Time-block*, but here the *Condition-variable* is varied for each *Experimental-trial*

To understand how these organizational annotation terms may be used, consider the following simple example study in which the participants perform two main tasks, each in two different task conditions. A researcher can organize this experiment in many ways including those described in Example 5 and illustrated schematically in Fig. 2.

- **Example 5: Three possible experiment designs for the simple study.**
- **Design 1** (left): Each *Recording* includes a single *Task* and *Condition-variable*, but has two *Time-block* sections separated by a relief break. Counterbalancing of *Task* and *Condition-variable* is done at the study level over four *Recordings* in different orders for each participant. *An Experimental-trial* includes three events.
- **Design 2** (center): Each *Recording* includes two *Time-block*s in which the participant performs one of the two main *Tasks*. Each main task *Time-block* comprises a single *Condition-variable*. *Task* and *Condition-variable* counterbalancing is performed across the time blocks within each recording.
- **Design 3** (right): Each *Recording* comprises one *Task* and continuous *Time-block*, but here the *Condition-variable* is selected at random for each *Experimental-trial*.

#### **A Sample Dataset Structure Viewer**

**Fig. 3:**
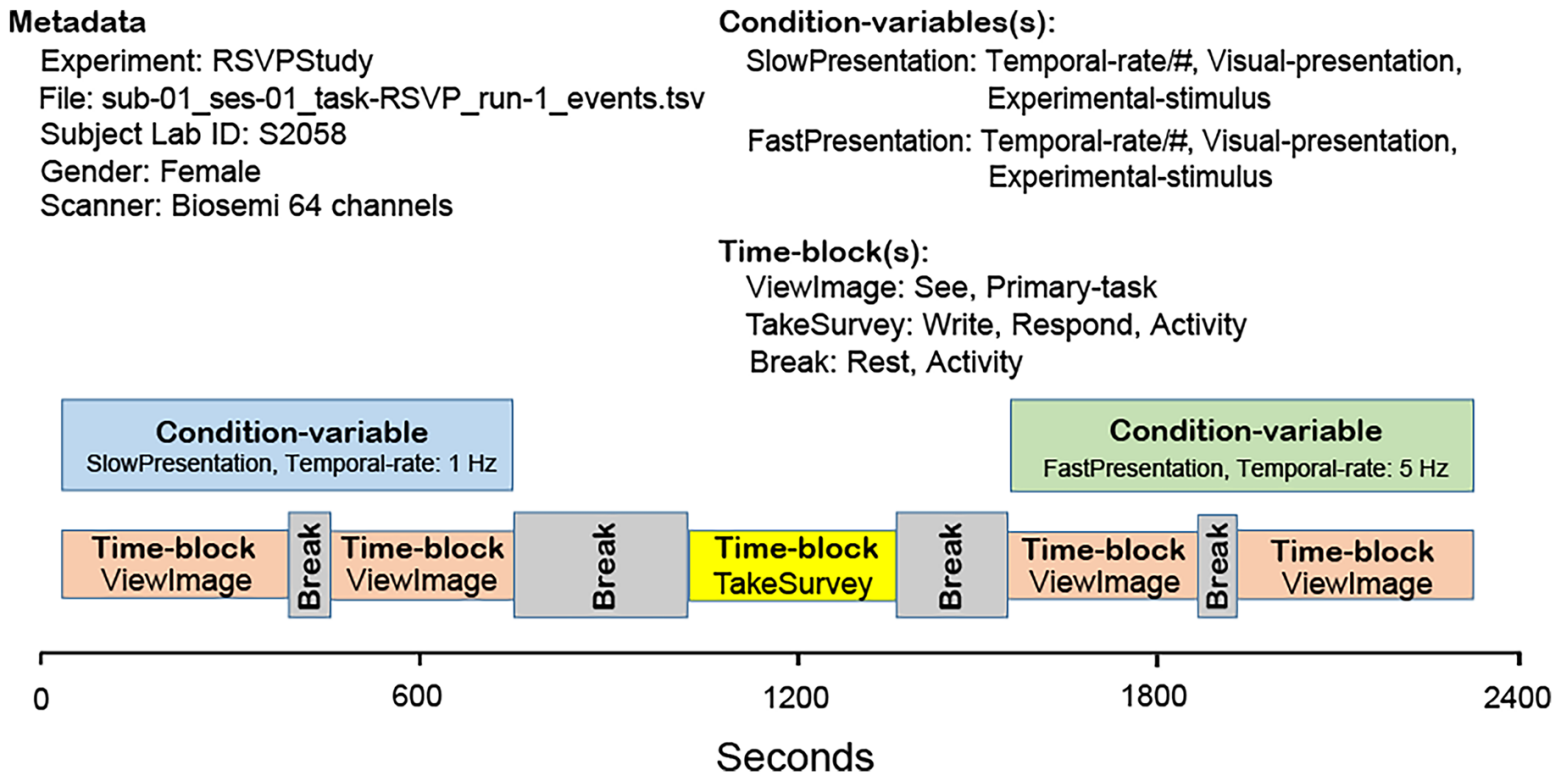
Mock-up of an experiment timeline automatically extracted from an event file annotated with *Task, Time-block,* and *Condition-variable* tags using a representation-dependent metadata extraction tool. Here *ViewImage* and *TakeSurvey* are user-defined *Time-block* defined names, while *SlowPresentation* and *FastPresentation* are user-defined *Condition-variable* type names. These defined terms are combined with *Onset* and *Offset* tags, enabling tools to automatically determine their placement on the visualized experiment timeline. The gaps in the timeline correspond to portions of the recording that are outside the temporal scope of an *Condition-variable* or a *Time-block* enduring event. Experiments typically have periods corresponding to relief breaks or changes in setup that are not annotated

A best practice for HED-3G tagging is to create *Definition* tags to represent the organization of the experiment, including definitions for each *Task*, *Condition-variable* and *Time-block* used in the study. These defined tags should then be grouped with *Onset* and *Offset* tags to mark where in the experiment the particular tagged aspect was in effect. Appropriate and consistent structural annotation can provide a wealth of information to automated data search and analysis tools. For example, a data repository could use this information to automatically produce a visualization of the dataset structure via a repository data browsing application. Figure 3 below shows a mock-up overview of such a visualization.

Such a timeline viewer application might be used by researchers to verify that the experiment was actually conducted according to the intended or documented specification. The availability of such annotations might also encourage researchers to more completely document items they might otherwise ignore or forget to tag (such as the administration of a survey between the two main task blocks). More details such as the presence of selected types of trial events might be optionally included in the lowest level of the timeline display when/if space permits.

Importantly, the organizational tags *Condition-variable* and *Time-block* make available information about changes in task and conditions at the supra-event level needed to inform any analysis, without requiring the annotator to include all their information when annotating every event during their time-span (see following paragraphs). Using these tags, automated tools could test whether there was a significant difference in some EEG measure across all available studies that included visual stimulus presentation conditions in which some control variable (e.g., stimulus rate) varied either within or across studies. One might also test across a set of HED-tagged datasets for subject traits or demographics that account for some feature variance (e.g., to test how available participant age may influence some measures of EEG dynamics or recorded behavior).

#### **Context-Aware Analysis**

To make effective use of the information provided by currently unfolding events, we are currently designing HED-3G analysis tools that perform tag remapping to document ongoing events that contribute to the active context of the intervening events. For example, suppose *PlayMovie* is an identifier defined to document the presentation of a short movie to the participant. A *(Def/PlayMovie, Onset)* event occurs at 20 s from the beginning of the file, and a *(Def/PlayMovie, Offset)* event occurs at 100 s. All the intervening events in the interval [20, 100] seconds should inherit the information that the specified movie clip is playing (and perhaps that the participant has been asked to view the movie with some specified task intent), without requiring the user to tag this information explicitly in the HED string for each such event. However, this mapping of the ongoing context should not anywise suggest that events occurring during the movie presentation should be associated with effects similar to those associated with the physical movie presentation onset and offset events.

HED-3G introduces the *Event-context* tag to capture this distinction. During analysis, compliant HED tools append a single *(Event-context, ….)* tag group to the HED string annotation of each annotated event*.* The tools then insert copies of the annotations of all then-ongoing enduring events into the *Event-context* tag group. Thus, an event occurring while the *PlayMovie* event is ongoing will have annotation including this information in its full-form *Event-context* tag group. This tag group may also hold many other types of information pertaining to the recording as a whole, as well as to the current task trial, block, and/or condition. While the actual mapping of an event’s context does not take place until the full-form annotation is assembled for analysis, the ability to use this facility for advanced analysis depends critically on the availability of appropriate annotations.

### HED Tools and Development Process

#### **User-Friendly Tagging Tools**

**Fig. 4:**
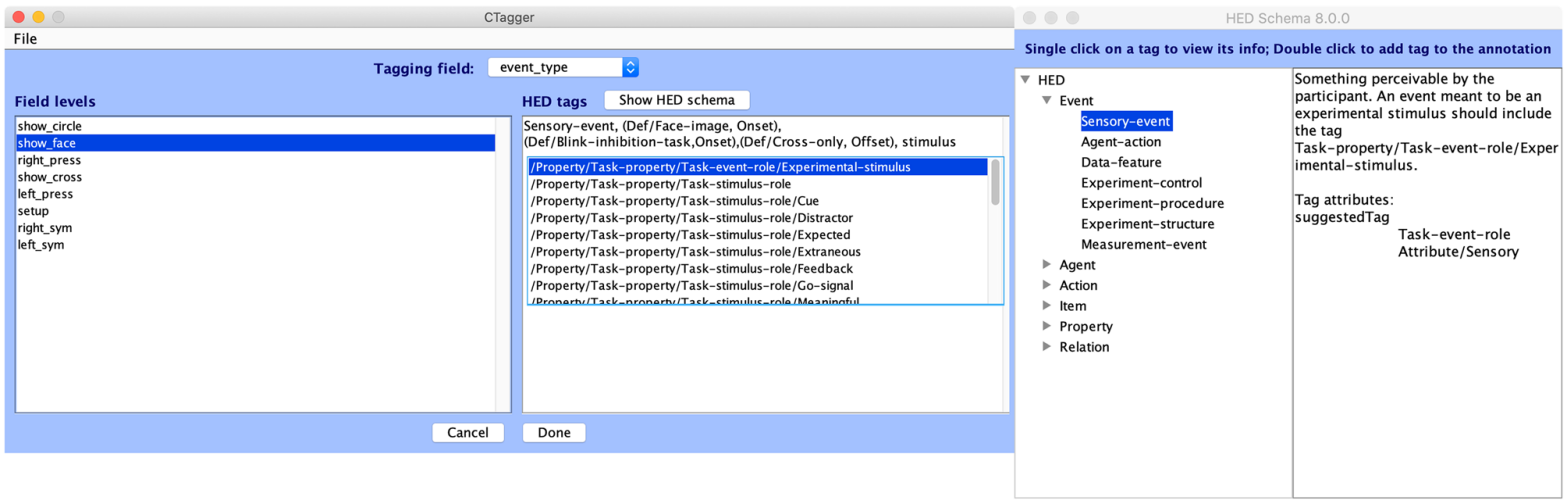
*CTagger GUI*. Users select event types on the left-side panel and compose HED strings on the right. The tool displays tags suggested by user inputs and provides a schema view from which users can browse and select tags to add to the event HED string

The original *CTagger* tool has been completely redesigned to enhance the ease of navigation during the annotation process as illustrated in Fig. 4.

The *CTagger* main interface consists of two parts: on the left, a list of event types to be annotated; on the right, a HED string input text area. *CTagger* suggests tags as users start typing, and users can also browse through an expandable tag view to select appropriate tags to add to the event string during tagging. The new *suggestedTag* and *relatedTag* attributes in the HED schema will be used to provide tagging hints for users during annotation.

#### **The HED Tool Libraries**

As discussed previously, compliant HED-3G analysis tools should handle the mapping of events to event context across the recording. The HED analysis tools also must convert all short-form tags to long form and expand defined tag terms into the tag groups they represent. Tool libraries in Python, Matlab, and JavaScript are under development to accomplish these expansions in easily callable formats. These libraries will provide a foundation for future tool development. Several other basic tools for searching and extracting time-locked data epochs are also already available or under development.

In addition to supporting common types of data search and collection operations, the structure of HED-3G may support future applications using more extensive knowledge-integration techniques including natural language processing. Future additions to the base HED schema could support inclusion of additional metadata into the HED schema, such as unique term identifiers and links to external resources and knowledge bases stored in external databases. Such links and identifiers, once created by domain experts, need not be visible during HED annotation and review. For example, there is a natural correspondence between HED schema elements and the Resource Framework Description (McBride, 2004) for interchange of web-linked data (Bigdely-Shamlo et al., 2016).

#### **Formalizing the Development Process**

In order to put the development processes on a firmer footing for community contributions, we have moved the code for all projects to the *hed-standard* GitHub organization site (https://github.com/hed-standard) and instituted the standard GitHub fork-pull-review-merge mechanism for proposing and incorporating schema changes and code updates. The *hed-schemas* repository (https://github.com/hed-standard/hed-schemas) holds all versions of the HED schemas including the library schemas. HED tools can download and cache any of these schema versions for use in validation and analysis. The base HED schema is stored in XML format for all machine processing purposes. The schema is also stored in a human-readable WYSIWYG MEDIAWIKI format to make it easier for developers to edit. Supported functions convert between MEDIAWIKI, XML, and JavaScript/HTML formats. A convenient JavaScript/HTML tool displays the schema in an interactive, expandable format in web browsers, facilitating schema search and review (Fig. 1). Issues, comments, and discussion are handled using the *Issues* mechanism of GitHub.

Other repositories housed on the *hed-standard* organization site include *hed-python* (validation and analysis tools as well as Docker containers for online deployment), *hed-javascript* (npm validation module called by BIDs for validating HED), *CTagger* (portable GUI tagging tools), and *hed-matlab* (HED validation and analysis tools as well as EEGLAB plug-ins). The HED project page is https://www.hedtags.org and all documentation is available at https://www.hed-resources.org.

## HED Now and Future: A Roadmap Forward

Much of the current design of HED-3G has benefitted from experience gained in performing a large, cross-study, HED-tag based mega-analysis (Bigdely-Shamlo et al., 2019a, b; Robbins et al., 2020) in which we learned not only what worked in HED-2G and what did not, but what questions we wanted to ask and couldn’t answer, as well as what approaches to more complete event descriptions might be most feasible.

We plan to formally release HED-3G in 2021, although HED and its supporting tools are available for download, review, comments, and contributions at the HED working group organizational website on GitHub (https://github.com/hed-standard). The current release of the BIDS validator (https://github.com/bids-standard/bids-validator) already has support for validation of both HED-3G and HED-2G annotated datasets, including tools for converting between short form and long form views of HED -3G tag strings and for basic HED string validation. Support for conversion between short and long form tagging in *CTagger* and enhancements to improve ease of annotation will also be included in the formal release. The release also includes a detailed specification document available online on the HED-Standard GitHub repository.

We have also re-released a HED-3G annotated version of the MEG/EEG components of a publicly-available multi-participant, multi-modal neuroimaging dataset on OpenNeuro (https://openneuro.org) under accession number *ds003645* along with an extensive case study in HED-3G annotation based on this dataset (Robbins et al., 2021). The data is from an experiment by Daniel Wakeman and Richard Hansen (Wakeman & Henson, 2015), originally shared under accession number *ds000117*.

We plan to complete implementation of infrastructure supporting some of the more advanced features of HED-3G such as definition processing and event duration mapping for studies archived in BIDS format after the initial release. Updating the current data search and analysis tools will follow. Documentation and support for library schema is also under development. More sophisticated task definition and event linkage annotation syntax, as well as support for annotating more complex spatial relationships, will be integrated into HED as soon as possible.

### Intermediate Goals

#### **Community Development**

HED development and HED coding of now and soon-to-be archived data must have substantial and sustained research community input and contribution to be successful. HED will not achieve its major aim of enabling meta/mega-analysis of the electrophysiological data and related time series accumulating in archives without adoption, active use, and exploitation, as well as creative further contributions by diverse communities of researchers and clinicians. To become involved in using and further developing HED, researchers must be convinced that annotation is an important part of assuring the legacy and increasing the total value of their data – both to their own research groups and to others.

#### **Library Schema**

Communities of researchers in areas such as clinical neurophysiology and music psychobiology have already expressed interest in developing discipline-specific HED vocabularies for EEG event annotation, and development of a SCORE library schema for terms used in clinical neurophysiology has already begun. Basic support for integrating schema libraries into the HED system are planned for the first release of HED-3G. However, much additional work needs to be done. Tools for building, documenting, versioning, and making available HED schema libraries all must function smoothly to be attractive for use in practice.

#### **Usability**

Having available open-source tools for performing useful analysis based on HED information as well as well-annotated HED-informed data archives linked to computing resources supporting the relevant tool libraries should increase interest in using HED. A careful, ambitious, and enthusiastic tutorial campaign will also be needed to allow HED annotation to become sufficiently widespread to reach “critical mass” momentum. Our experience has taught us that performing truly useful data annotation is not trivial, even given good tools and tutorials. Development of supporting tools for assisting in annotation is ongoing and critical for good annotation. We have implemented various (“Wizard”) guide systems for setting up and annotating experiments. However, the HED system itself has evolved more rapidly than the applicable tools. Thus, much work needs to be done in this area. Better visualizations and graphical user interfaces to underlying tools are needed for all phases of the HED life-cycle: experiment, annotation, review, and analysis.

#### **BIDS Metadata and COBIDAS**

The BIDS standards group has an active ongoing effort to better integrate Committee on Best Practices in Data Analysis and Sharing (COBIDAS) recommendations into the BIDS specification (Nichols et al., 2017). This effort includes enhancing BIDS requirements and recommendations for documentation as well as provided standardized templates for helping users incorporate needed information. The HED *Recording* and *Metadata* tags provide infrastructure for building tools that automatically extract standardized metadata from a BIDS study and then insert the extracted information into the context for each data recording to enable analysis. Such tools would greatly facilitate cross-study analysis. For example, one could determine (in an automated fashion) whether demographic metadata such as age or gender are significant factors in accounting for subject differences in some physiological or behavioral measure of interest? If so, in what types of tasks?

#### **Analysis Tools**

EEGLAB tools currently incorporate HED as a foundation to support analysis and re-analysis of individual studies as well as meta/mega-analysis of archived data across studies. Other MATLAB tool environments such as Fieldtrip (Oostenveld et al., 2011) may be able to easily incorporate handling of HED tag information by using or adapting available HED MATLAB library functions. We already have Python and MATLAB libraries for transforming between long and short form annotations. More comprehensive libraries for expanding annotations and for sophisticated searching are under being refactored to support HED-3G. The validation and other tools are available as online on the *hedtools* website (https://hedtools.ucsd.edu/hed). These tools are implemented in a Docker container with detailed deployment instructions. We also plan to make these online tools available as BIDS apps (Gorgolewski et al., 2017a).

#### **An integrated Data, Tools, and Compute Resource**

We have begun the process of enlarging the HED user community and annotating studies in HED-3G for archiving, retrieval and computation via *NEMAR* (https://nemar.org), a DATCOR (integrated data, tools, and compute resource) for human electrophysiological data that we and collaborators are now building. NEMAR will also act as a portal to *OpenNeuro* (https://OpenNeuro.org), the NIMH-supported archive for human neuroimaging data of all modalities (Gorgolewski et al., 2017a, b). The EEGLAB computational portal to the XSEDE high-performance computing network (Martínez-Cancino et al., 2020) via the Neuroscience Gateway (Sivagnanam et al., 2020), soon to be integrated with *NEMAR*, will allow intensive, high-performance processing of HED-tagged BIDS-organized data without requiring voluminous data transfer and data copy management. As part of Standardized processing pipelines for *NEMAR* are being developed.

#### **Other Time Series Modalities**

While our own research has focused on analysis of scalp EEG data, the HED system is equally applicable to any human neuroimaging experiment, and immediately to experiments using MEG, iEEG, fNIR, or (equally) fMRI. HED library schema extending the top-level HED schema vocabulary to include modality-specific terms, for instance for body and eye movement tracking data used in Mobile Brain/Body Imaging (MoBI) paradigms should be straightforward (though not simple) to build and integrate (Makeig et al., 2009). Future uses for HED annotation need not be restricted to neuroimaging – any time series or time sequence in which timing of events is recorded could be able to be usefully represented in HED using appropriate library schema.

### Open Questions

Although HED-3G represents a significant advance in the annotation of events for meaningful analysis, several open questions and long-term development tasks remain in addition to ongoing discussion and maintenance of the base HED vocabulary and library schemas.

#### **Documenting Task-Event Relationships**

Often in human neuroimaging, and particularly in human EEG/MEG experiments, the participant *Task* rather than the stimulus sequence varies across conditions. For example, in one condition, the participant is asked to respond with a button press only to one type of stimuli, while in another condition the participant is to respond only to some other type of stimuli. Here the stimulus presentation parameters themselves do not change between conditions. A complete record of events in an experiment should capture both the detailed intentions and expectations of the subject (as specified in subject task instructions) as well as the stimulation details (as produced by the experiment control application).

HED-3G can express what a subject actually did (e.g., ‘pressed the red button’) but does not yet have good semantics for expressing complex relationships and causal linkages between events mediated by the structure of the user task. For example, simply labeling one stimulus type as a *Target* is not sufficient and should be related more explicitly to the particular task the participant was performing.

A mechanism must also be developed for annotating linkage between events when their conditional linkage involves a set of rules. This process is difficult even for well-known tasks such as the *N*-back continuous performance task (Kirchner, 1958) in which a subject is to indicate whether each current stimulus matches the one presented *N* stimuli earlier. A solution we are now exploring is to develop a task specification meta-code to enable comparison of events on the basis of their function and value in the context of the task.

The original plan during development of HED-1G and HED-2G was to incorporate the CogPO (Turner & Laird, 2012) list of task paradigms, with hopes of linking HED event descriptions to task databases such as the *Cognitive Atlas* (https://www.cognitiveatlas.org). We removed the HED-2G *Paradigm* tags from HED -3G, however, because the available paradigm nomenclature is not standardized. Text descriptions of tasks in the *Cognitive Atlas* vary in specificity and use only broadly-defined and sub-field specific terminology. These descriptions do not, at present, represent machine-actionable information. Nonetheless, associating a dataset with a well-known paradigm detailed in the *Cognitive Atlas* or elsewhere remains possible in HED-3G using various informational tags. HED-3G specifically has *Property/Informational-property/Metadata/CogAtlas/#* and *Property/Informational-property/Metadata/CogPo/#* tags. However, merely associating these respective IDs with the recording does not present task information in a machine-actionable form. Currently, HED-3G users can define a name representing a *Task* and associate tags that specify concepts relating to the task (essentially, listing keywords pertaining to it). This approach is not a true answer to task specification, though it may allow searching across studies for task keywords of interest.

#### **Immediate Context**

The subject of temporal relationships between events exposes deeper neurological questions. Both brain and behavioral dynamics are shaped not only by intentions but also by prevailing expectations, including those created by immediately preceding events. Defining *context neighborhoods* of influence of preceding events of interest and then using this information in automated tools is likely possible to implement, but work to build the necessary infrastructure and test its utility for analysis is just beginning.

#### **Automating Annotation**

Another long-term goal is to deploy more of the tagging process in earlier stages of execution, particularly by making generation of HED tags for stimulation events an active responsibility of experiment control applications. We hope to work with major control program maintainers to add this option. It might also be possible to build some automated tagging facility to capture the logic of the experiment control program, including intended functional relationships between delivered stimulus events and intended participant motor action events (e.g., Push the button whenever you see a red square…). Development of a way to map control programs into “meta-scripts” would facilitate the incorporation of HED annotations of task structure without requiring programming knowledge and careful construction by experimenters. Unfortunately, information gathered from the event log itself would yield only stochastic information (control rules with some degree of uncertainty), and the tools required would be control-program specific. Thus, HED will need a meta-language system for specifying task design that is simple enough for any investigators to learn and use easily.

#### **Spatial Relationships**

For nearly 50 years, and still today, the most common setup for EEG experiments has been for the participant to sit facing with eyes fixated on the center of a computer monitor on whose 2-D surface some collection of 2-D visual stimuli are presented. Although the number of parameters needed to fully specify the spatial embedding of this experience are relatively few (i.e., distance from screen to eyes, size and position of each stimulus on the screen), recording such information as an integral part of data recording has not been standard. New graphics tools to easily measure stimulus size and position, and apps using new cellphone 3D scanners can make this documentation routine. Specifying the spatial relationship of participants to perceived events in experiments that use 3-D movies or virtual displays and include eye, head, or full body movements in real life settings is a further neuroinformatics frontier.

### The View Ahead

Although human electrophysiological data in the form of scalp EEG was the first noninvasive human brain activity recording modality, dating from Hans Berger circa 1926 (İnce et al., 2020), progress in EEG analysis and interpretation has long lagged behind technical developments for its acquisition. In the clinical neurophysiology field, visual inspection of the raw channel records is still the most prevalent mode of information extraction, while in cognitive neuroscience, study of details in event-related response averages across classes of similar events in single or spatially averaged scalp channel signals has long dominated practice and teaching.

While substantial progress has been made in the past twenty years toward extracting a rich spectrum of information about human brain dynamics contained in electrophysiological recordings (EEG, MEG, iEEG), much data collected during this period has not been mined using now freely-available analysis approaches. Further, applications of machine learning to electrophysiological data are still in their infancy and require availability of well-annotated data to deliver accurate markers and new understanding of how the brain supports human behavior and experience, both normal and pathological.

We believe that, given sufficient care, interest, and continued investment, the HED system can, should, and will play an important role in this evolution. Further expansion of HED annotation to many types of time series and time-ordered data also appears a potent possibility. The overall goal of the HED development effort remains — to make time series data preserved, archived, and shared under the FAIR principles readily useful for both immediate and future analysis, interpretation, and understanding. HED will enable well-annotated datasets to be searched, summarized and extracted from at a granularity not available under current systems (Findable). All of HED development is open source and freely available (Accessible). The HED design and planned future enhancements will allow easy integration into computational and archival platforms (Interoperable). Further, for electrophysiological or nearly all time-series data sets, detailed event annotations are essential for analysis (Reusable).

#### **Becoming Involved**

We encourage interested researchers to become involved in HED development and standardization. Questions and suggestions should be directed to the issues forum of the *hed-specification* and other repositories hosted at https://github.com/hed-standard. Online HED tools are available at https://hedtools.ucsd.edu/hed. The HED-3G specification document is open for comments. Users interested in participating in developing a library schema for a particular research field or sub-field should communicate their interest by posting on the issues forum of the *hed-schemas* repository (https://github.com/hed-standard/hed-schemas).

## Information Sharing Statement

All material is open-source and available at the *hed-standard* organization GitHub repository: https://github.com/hed-standard*.* Online tools are available at https://hedtools.ucsd.edu. A fully HED annotated example dataset is available on OpenNeuro (https://openneuro.org as *ds003645*.

## Data Availability

All code is available at the *hed-standard* working group organization GitHub site: https://github.com/hed-standard*.*
