# Correction to: Building FAIR Functionality: Annotating Events in Time Series Data Using Hierarchical Event Descriptors (HED)

**DOI:** 10.1007/s12021-023-09628-4

**Published:** 2023-04-11

**Authors:** Kay Robbins, Dung Truong, Alexander Jones, Ian Callanan, Scott Makeig

**Affiliations:** 1 Department of Computer Science, University of Texas At San Antonio, San Antonio, USA; 2 Swartz Center for Computational Neuroscience, Institute for Neural Computation, University of California San Diego, San Diego, USA

**Correction to: Neuroinformatics (2021) 20:463-481** **https://doi.org/10.1007/s12021-021-09537-4**

The original version of this article unfortunately contained typesetting errors. The texts in Examples 1, 2, 3, and 4 were captured incorrectly. The correct presentation of these examples are shown below.

**Example 1. A first-generation HED annotation** of a presentation of a visual stimulus consisting of both a red triangle and a green square:



**Example 2. Second-generation HED annotation** for a visual stimulus presentation of a red triangle and a green square. [Note that, here and below, text in square brackets is didactic commentary, not HED syntax.]



**Example 3. Full long form and compact short form** of a HED-3G annotation of the same event as in Examples 1 and 2, in which a red triangle and a green square are shown to the participant. Again, stimulus size, duration and positioning details are here omitted for brevity. The text in square brackets is commentary and not part of the tag string.



**Example 4. Define ScreenSetup to represent the experimental setup used to present visual stimuli.**



The original article has been corrected.

